# Preoperative assessment of peripheral vascular invasion of pancreatic ductal adenocarcinoma based on high-resolution MRI

**DOI:** 10.1186/s12885-023-11451-8

**Published:** 2023-11-10

**Authors:** Xiaoqi Zhou, Danyang Xu, Meng Wang, Ruixia Ma, Chenyu Song, Zhi Dong, Yanji Luo, Jifei Wang, Shi-Ting Feng

**Affiliations:** Department of Radiology, The first Affiliated Hospital, Sun Yat-Sen University, 58th, The Second Zhongshan Road, Guangzhou, Guangdong China

**Keywords:** High-resolution-MRI, CT, Pancreatic ductal adenocarcinoma, Vascular invasion

## Abstract

**Objectives:**

Preoperative imaging of vascular invasion is important for surgical resection of pancreatic ductal adenocarcinoma (PDAC). However, whether MRI and CT share the same evaluation criteria remains unclear. This study aimed to compare the diagnostic accuracy of high-resolution MRI (HR-MRI), conventional MRI (non-HR-MRI) and CT for PDAC vascular invasion.

**Methods:**

Pathologically proven PDAC with preoperative HR-MRI (79 cases, 58 with CT) and non-HR-MRI (77 cases, 59 with CT) were retrospectively collected. Vascular invasion was confirmed surgically or pathologically. The degree of tumour-vascular contact, vessel narrowing and contour irregularity were reviewed respectively. Diagnostic criteria 1 (C1) was the presence of all three characteristics, and criteria 2 (C2) was the presence of any one of them. The diagnostic efficacies of different examination methods and criteria were evaluated and compared.

**Results:**

HR-MRI showed satisfactory performance in assessing vascular invasion (AUC: 0.87–0.92), especially better sensitivity (0.79–0.86 vs. 0.40–0.79) than that with non-HR-MRI and CT. HR-MRI was superior to non-HR-MRI. C2 was superior to C1 on CT evaluation (0.85 vs. 0.79, *P* = 0.03). C1 was superior to C2 in the venous assessment using HR-MRI (0.90 vs. 0.87, *P* = 0.04) and in the arterial assessment using non-HR-MRI (0.69 vs. 0.68, *P* = 0.04). The combination of C1-assessed HR-MRI and C2-assessed CT was significantly better than that of CT alone (0.96 vs. 0.86, *P* = 0.04).

**Conclusions:**

HR-MRI more accurately assessed PDAC vascular invasion than conventional MRI and may contribute to operative decision-making. C1 was more applicable to MRI scans, and C2 to CT scans. The combination of C1-assessed HR-MRI and C2-assessed CT outperformed CT alone and showed the best efficacy in preoperative examination of PDAC.

**Supplementary Information:**

The online version contains supplementary material available at 10.1186/s12885-023-11451-8.

## Introduction

Pancreatic ductal adenocarcinoma (PDAC) is a highly aggressive malignant tumour with a 7–9% 5-year survival and the seventh highest mortality rate worldwide [1, 2]. At the time of diagnosis, most PDAC patients are diagnosed at a late stage, thus losing the chance of radical resection due to involvement of peripancreatic structures or distant metastases [3, 4].

Vascular invasion is of great clinical importance because it is a predictor of the prognosis and survival of patients with PDAC [5, 6]. Moreover, it is an important factor influencing therapeutic options [7]. Currently, radical surgical resection is the best treatment for PDAC [8]. The National Comprehensive Cancer Network (NCCN) guidelines classify PDAC into resectable, borderline resectable, and unresectable according to the tumour–vascular vessel relationship [9]. The vessels in need of assessment were divided into the arterial system (celiac trunk, common hepatic artery, superior mesenteric artery, and splenic artery) and venous system (portal vein/superior mesenteric vein). In cases of PDAC with arterial invasion, it is usually difficult to achieve R0 resection and may require neoadjuvant chemotherapy to reduce the extent of tumour invasion. Venous resection and reconstruction are the options for patients with limited venous invasion. However, vascular invasion requires surgical and pathological confirmation and may result in unnecessary surgical trauma in patients with unresectable PDAC. Therefore, the preoperative determination of vascular invasion is critical for the selection of therapeutic options and surgical planning.

Preoperative non-invasive imaging evaluations, especially Computed tomography (CT), have been widely used in clinical practice because of their convenience and high resolution [10, 11]. CT plays an important role in the evaluation of vascular invasion in PDAC with high spatial resolution. In most institutions, presurgical staging of PDAC relies on dedicated high-quality multidetector row CT of the pancreas, which is recommended as a primary diagnostic test by the NCCN guidelines [9, 12, 13]. However, the accuracy of CT is 77% for predicting resectability and 93% for predicting unresectability, indicating the need for improvement [14, 15]. In recent years, the gradual development and application of MRI have shown unique advantages in detecting and diagnosing small PDAC lesions and metastases [16, 17]. Dynamic Magnetic resonance imaging (MRI) has been reported to have equal or better specificity than helical CT for determining local tumour extension and vascular involvement [18]. However, the diagnostic efficacy of CT and MR for vascular invasion remained inconclusive [19, 20]. With the advancement of MRI technology, high-resolution MRI (HR-MRI) provides important information for the assessment of intravascular lesions and the relationship between lesions and vessels, significantly improving diagnostic accuracy and assessment efficacy [21, 22]. Moreover, gadobutrol is a high-concentration extracellular macrocyclic gadolinium-based contrast agent that provides significantly higher mean signal-to-noise ratios in large and small vessels and improves the contrast between blood vessels and the surrounding tissue [23, 24]. Thus, HR-MRI combined with gadobutrol may provide better image quality than conventional MRI. The presence of vascular invasion criteria on imaging used by the NCCN guidelines includes tumour-vascular contact > 180°, vessel narrowing, or contour irregularity [9]. However, because of the different capabilities of CT and MR, it is unclear whether these methods share the same set of evaluation criteria.

This study aimed to evaluate the combination of HR-MRI and gadobutrol in the assessment of PDAC vascular invasion, compare its accuracy with that of non-HR-MRI and CT, and clarify the applicable diagnostic criteria for CT and MRI.

## Materials and methods

### Patient selection

The study protocol was approved by the Institutional Review Board, and the requirement for informed consent was waived (approval number: [2021] 025). All procedures were carried out in accordance with the approved guidelines.

**Fig. 1:**
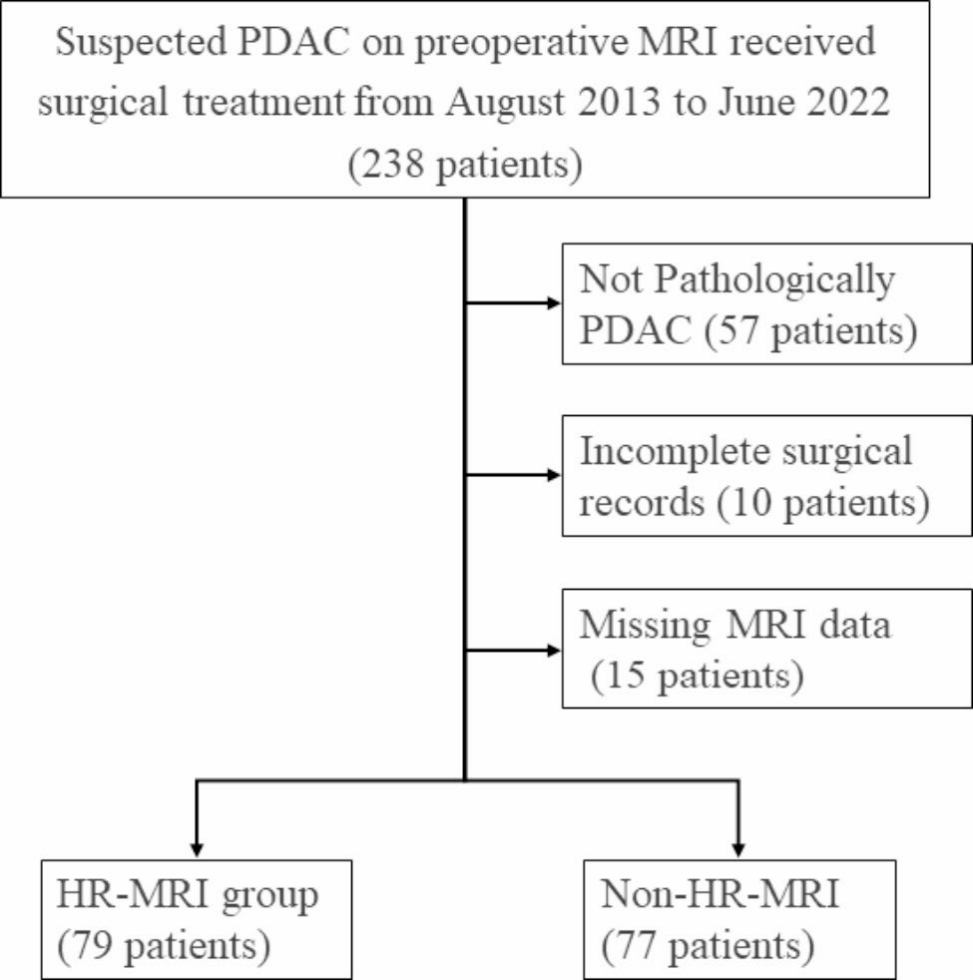
Flow diagram of study population

A flowchart of the data collection and study design is shown in Fig. 1. This retrospective analysis included patients who underwent enhanced MRI within 1 month before radical surgery for PDAC without neoadjuvant therapy between August 2013 and June 2022 at The First Affiliated Hospital, Sun Yat-Sen University. The exclusion criteria were as follows: (1) poor MR quality and (2) missing information on tumour and vascular relationships in the surgical records. Ultimately, 79 patients who underwent HR-MRI (58 with CT) and 77 who underwent non-HR-MRI (59 with CT) were enrolled. Group 1 consisted of 79 h-MRI cases, and group 2 included 58 corresponding CT cases of group 1. Similarly, group 3 included 77 non-HR-MRI cases, and group 4 included 59 corresponding CT cases of group 3.

### Imaging protocol and image analysis

All patients in the HR-MRI group underwent gadobutrol-enhanced abdominal MRI using a 3.0-T system (GE SIGNA Pioneer, GE Medical Systems, Milwaukee, WI, USA) with unenhanced imaging (in-phase and opposed-phase T1-weighted images; T2-weighted images; high-resolution T2-weighted black blood sequence with and without fat suppression; and diffusion-weighted images) and contrast-enhanced T1-weighted imaging. The non-ionic gadolinium-based contrast medium (Gadobutrol, Gadovist, Bayer Schering Pharma, Germany) at a dose of 0.1 mmol/kg was injected utilizing a power injector at a rate of 1.5 mL/s followed by a 30 mL saline flush. The specific parameters of each sequence are listed in Table 1. The HR-MRI consists of all these sequences, especially the high-resolution T2-weighted black blood sequence and field of view optimized and constrained undistorted single-shot diffusion-weighted images (DWI) with the slice thickness of 3 mm.

**Table 1:** HR-MRI scan sequence and parameters

Sequences	TR(ms)	TE(ms)	FOV(cm)	Scan matrix	Slice thickness (mm)	Slice gap (mm)	Flip angle (°)	Number of excitations	Fat saturation	Orientation	category	mode
T1WI IN/OUT	5.5	1.8	34	320 × 288	3	-1.5	12	1	Yes	Axial	IDEAL	3D
T2WI	10,588	85.8	34	320 × 320	6	1.0	160	1	No	Axial	FSE	2D
High-resolution T2WI	11,250	85.9	32	320 × 320	3	0	111	4	No	Axial	FSE	2D
High-resolution T2WI-FS	6316	85.9	32	320 × 320	3	0	111	4	Yes	Axial	FSE	2D
SSFSE	900	70.8	44	384 × 256	8	10	180	10	No	Coronal	FSE	2D
Focus DWI	8677	64.2	38	160 × 64	3	0	90	2/16	Yes	Axial	EPI	2D
DWI*	5455	77.2	36	128 × 128	6	0.6	90	2/4	Yes	Axial	EPI	2D
DISCO	5.7	2.1	38	288 × 256	3	-1.5	12	1	Yes	Axial	DISCO	3D
Cor DISCO	5.7	2.1	38	288 × 256	1.5	-1.5	12	1	Yes	Coronal	DISCO	3D

TR, repetition time; TE, echo time; FOV, field of view; WI, weighted imaging; FS, fat-suppressed; Focus, field of view optimized and constrained undistorted single-shot; DWI, Diffusion-weighted imaging; SSFSE, single-shot fast spin echo; DISCO, Differential Subsampling with Cartesian Ordering

*b factors = 50 and 800 s/mm^2^

For non-HR-MRI, the patients underwent normal gadolinium-enhanced abdominal MRI using 1.5-T system (GE Brivo MR355, GE Medical Systems, Milwaukee, WI, USA) or 3.0-T systems (MAGNETOM Verio, MAGNETOM Prisma, MAGNETOM Vida; Siemens Healthcare, Erlangen, Germany). Since most of the patients in this group were examined on the MAGNETOM Prisma, the examination parameters of this scanner are listed in Supplementary Table 1. Some patients were examined using a multislice spiral CT scanner (Aquilion 64, Canon Medical System) with the following CT scanning parameters: craniocaudal plane; slice thickness, 0.5 mm; slice increments, 0.5 mm; pitch, 0.9; tube voltage, 120 kV; tube current, 20 mAs; collimation, 64 × 0.5 mm. An iodinated contrast agent (iopromide, Ultravist, Bayer Schering Pharma, Germany) was injected into the antecubital vein at a rate of 3.5mL/s using an automatic injector, followed by 40 mL of saline at the same rate. Enhanced images were acquired in the arterial phase (35-s delay) and venous phase (65-s delay).

All MR and CT images were retrospectively reviewed by two abdominal radiologists with 6 and 9 years of experience, respectively, who were blinded to the pathological and clinical data of all patients. Conclusive discrepancies were finally discussed and resolved by mutual consensus after joint reassessment of the images. Tumour location was defined as the head/neck and body/tail of the pancreas, according to the spatial relationship between the tumour and the superior mesenteric vein. The tumour size was measured on axial images using the longest cross-sectional dimension. The relationships between the tumour and celiac trunk artery (CA), hepatic artery (HA), superior mesenteric artery (SMA), splenic artery (SpA), portal vein-superior mesenteric vein (PV-SMV), and gastroduodenal artery (GDA) were recorded. The analysis included circumferential tumour contact (less than or equal to 180° versus more than 180°), vessel narrowing (change in the vessel calibre) and contour irregularity (interruption or obstruction of vessel wall). MR evaluation is based on all axial and coronal images. However, the conventional MRI did not have coronal scan images. In conventional MRI group, we obtained the coronal images by MPR based on dynamic volumetric enhanced axial images with a slice thickness of 2 mm. CT evaluation is based on axial and multiplanar reformation images. In MRI, tumour composition is determined by combining T2 signal, enhancement and DWI restriction to determine tumour boundary, while in CT, tumour boundary is mainly determined by density and enhancement difference. Additionally, the variant anatomy of the vessels was identified before confirming the peripancreatic structures.

The invasion of each vessel was determined using two criteria. Criteria 1 (C1): (1) circumferential tumour contact > 180°, (2) vessel narrowing, and (3) contour irregularity. Criteria 2 (C2): (1) circumferential tumour contact > 180°, (2) vessel narrowing, or (3) contour irregularities. The accuracies of different criteria and examination methods were compared.

### Clinical and pathological analyses

The results of tumour-vessel contact explored by the surgeon in the surgical record and the results in the pathology report were considered the gold standard in this study. Other clinical information, including patient demographics and surgical procedures, were also examined. Surgical procedures were classified as pancreatoduodenectomies, partial pancreatectomies (including distal pancreatectomies, partial pancreatectomies, and tumour resections), or exploratory laparotomies.

### Statistical analysis

SPSS (version 25.0; IBM Corp., Armonk, NY, USA) and R (version 4.2.2) were used for the analysis. Continuous variables were evaluated using the Kolmogorov–Smirnov normality test. Means and standard deviations (SDs) were used to describe the continuous variables. An independent samples t-test was used to analyse continuous variables. Categorical variables were analysed using the χ2 or Fisher’s exact test.

The interobserver agreement of tumour-vessel relationship was evaluated by interclass correlation coefficients (ICCs) for all readers and weighted kappa coefficients for different readers. The interobserver agreement was graded as follows: 0–0.20, slight; 0.21–0.40, fair; 0.41–0.60, moderate; 0.61–0.80, substantial; and 0.81–1.00, almost perfect.

Receiver operating characteristic (ROC) curves were used to evaluate the predictive efficacy of each group and the criteria of sensitivity (Sen), specificity (Spe), and area under the ROC curve (AUC). The Delong test was used to compare the accuracy of the different evaluation methods. All differences were considered statistically significant at *P* < 0.05. False discovery rate (FDR) was used to correct the *P* value and obtained the *Q* value(p.adjust).

## Result

### Patient demographics and lesion characteristics

A total of 79 patients who underwent HR-MRI (58 with CT) and 77 without (59 with CT) were enrolled in this study. The clinical information of both groups is shown in Table 2.

**Table 2:** Clinical information

Characteristic	HR-MRI (n = 79)	non-HR-MRI (n = 77)		*P*
Age (year), mean ± SD (range)	58.84 ± 1.21	63.54 ± 1.20	-2.77	0.006
Sex				
Male	55	48	0.73	0.39
Female	24	29		
Tumour location in the pancreas				
Head/neck	50	53	0.72	0.40
Body/tail	29	23		
Maximal tumour diameter (mm), mean ± SD (range)	30.06 ± 1.88	29.88 ± 1.38	0.079	0.94
T stage				
1c	15	11	1.33	0.72
2	49	53		
3	11	8		
4	4	5		
N stage				
x	4	7	1.28	0.73
0	38	38		
1	30	27		
2	7	5		
Type of operation				
Pancreatoduodenectomy	57	57	0.23	0.89
Partial pancreatectomy	17	14		
Exploratory laparotomy	5	5		
Vascular invasion				
Arterial	12	8	0.80	0.37
Venous	14	18	0.84	0.36

### Sensitivity and specificity of prediction of vascular invasion based on different radiological assessment criteria

The interobserver ICC ranged from 0.83 to 0.92 when assessing tumour-vessel relationship. The interobserver differences between different groups and vessels is presented in Table 3.

**Table 3:** Interobserver agreement of tumour-vessel relationship

Vessels		All Readers	Reader-1 vs. Reader-C	Reader-2 vs. Reader-C	Reader-1 vs. Reader-2
Group1	V + A	0.87	0.76	0.97	0.72
	V	0.89	0.78	0.97	0.75
	A	0.86	0.75	0.95	0.71
Group2	V + A	0.91	0.81	0.97	0.78
	V	0.92	0.82	0.98	0.80
	A	0.91	0.82	0.98	0.79
Group3	V + A	0.83	0.74	0.96	0.69
	V	0.85	0.73	0.97	0.70
	A	0.92	0.77	0.98	0.77
Group4	V + A	0.91	0.83	0.98	0.80
	V	0.90	0.78	0.96	0.73
	A	0.92	0.80	0.98	0.78

Group 1, HR-MRI group; Group 2, CT of HR-MRI group; Group 3, non-HR-MRI group; Group 4, CT of non-HR-MRI group; A, artery; V, venous; Reader-1, a radiologist with 6 years of pancreatic experience; Reader-2, a radiologist with 9 years of pancreatic experience; Reader-C, mutual consensus after joint reassessment. All *P* < 0.001

All MR and CT methods had good specificity, but their sensitivity varied widely. HR-MRI showed the highest overall AUC value for all vessel assessments under both criteria, whereas non-HR-MRI showed the worst overall AUC value. Non-HR-MRI was less sensitive than CT and HR-MRI for both the C1 and C2 criteria, especially in assessing arterial invasion, with a sensitivity of only 40%.

In the assessment of arterial invasion, all methods showed a high specificity of > 97%, which was better than that of the venous assessment. HR-MRI showed the highest sensitivity and specificity for assessing arterial invasion regardless of the C1 or C2 criteria. In the assessment of venous invasion, CT had the best specificity of up to 98% with the C1 criteria, but a lower sensitivity than HR-MRI.

**Fig. 2:**
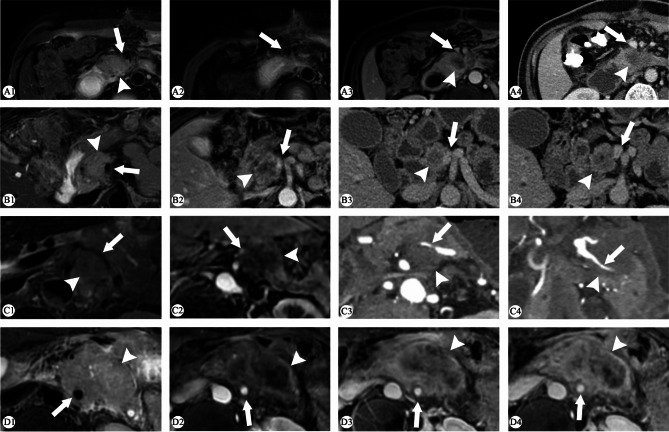
Cases in HR-MRI group. **A1–A4**, a case of pancreatic ductal adenocarcinoma (PDAC) located in the pancreas uncinate process (white arrowhead). High-resolution T2 weighted imaging (WI) showed the irregular vascular wall of superior mesenteric vein (SMV) (white arrow) (A1–A2) and contract enhanced (CE)-T1WI showed vascular stenosis (A3). The circumferential tumour contact was less than 180° without significant abnormalities in the vessel wall on CT (A4). Vascular invasion was judged to be positive on MRI based on both C1 and C2 criteria, while CT results to be negative. SMV invasion was confirmed during pancreaticoduodenectomy. **B1-B4**, a case of PDAC located in the pancreatic head (white arrowhead). The circumferential tumour contact of SMV was > 180° on MRI but < 180°on CT, without significant abnormalities in the SMV wall (white arrow). Only C2 criteria of CT determined the presence of vascular invasion. SMV invasion was confirmed during pancreaticoduodenectomy. **C1–C4**, a case of PDAC located in the pancreatic body (white arrowhead). High-resolution T2WI and CE-T1WI showed the narrowed lumen of SpA surrounded by tumour, and the wall is ill-defined (white arrow) (C1–C2). Axial and oblique coronal CT also showed circumferential tumour contact over 180° and narrowed vascular wall (C3–C4). All criteria on both CT and MRI determined positive vascular invasion. SpA invasion and peritoneal implant metastasis was confirmed during exploratory laparotomy. **D1–D4**, a case of PDAC located in the pancreatic body/tail (white arrowhead) with MRI. High-resolution T2WI and CE-T1WI showed circumferential tumour contact of > 180° of superior mesenteric artery (SMA) (white arrow), but without obvious narrowing or irregularity. SMA invasion was negative according to C1 but positive according to C2 criteria. SMA invasion was confirmed during exploratory laparotomy and Nanoknife tumour ablation

**Fig. 3:**
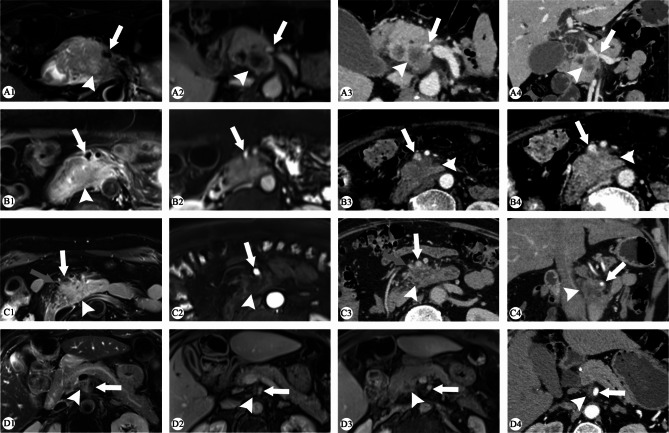
Cases of non-HR-MRI group. **A1–A4**, a case of pancreatic ductal adenocarcinoma (PDAC) located in the pancreatic head (white arrowhead). MRI showed the circumferential tumour contact of SMV was less than 180°, without obvious narrowing (white arrow). The vessel wall on the contralateral side of the tumour does not appear to be polished, but it may be caused by volume effects (A2). Axial and oblique coronal CT showed the circumferential tumour contact of SMV was less than 180° with local narrowing (A3–A4). SMV invasion was considered positive on CT but negative on MRI according to both criteria. Venous invasion was confirmed during pancreaticoduodenectomy. **B1–B4**, a case of PDAC located in the pancreatic head (white arrowhead). The circumferential tumour contact was 180° with irregular vessel wall on T2 weighted imaging (WI) (white arrow) (B1), with vascular narrowing on CE-T1WI (B2). The circumferential tumour contact was over 180° with irregular vessel lumen on CT (B3–B4). All criteria on both CT and MRI determined positive SMV invasion. The tumour was found to be tightly packed but separable from the SMV during pancreaticoduodenectomy. **C1–C4**, a case of PDAC located in the pancreatic head (white arrowhead). The circumferential tumour contact of SMA was over 180° with irregular vessel wall on T2WI and CE-T1WI (white arrow) (C1–C2), while the contact was over 180° with irregular vessel lumen on axial and oblique coronal CT (C3–C4). The circumferential tumour contact of SMV was less than 180° on MRI and CT (gray arrow) (C1, C3). All criteria on both CT and MRI determined positive SMA invasion and negative SMV invasion. SMV and SMA invasion was confirmed during exploratory laparotomy. **D1–D4**, a case of PDAC located in the pancreatic neck (white arrowhead) close to the right side of SMA. MRI and CT showed the circumferential tumour contact of SMA was less than 180°, without obvious narrowing or irregularity (white arrow). All criteria on both CT and MRI determined negative SMA invasion. The tumour was tightly adherent to the right side of SMA and could only be sharply separated during pancreaticoduodenectomy

Compared with the C1 criteria, the C2 criteria improved the sensitivity of vascular assessment to some extent while compromising the specificity. The C2 criteria was not inferior to C1 in the overall AUC value, except for a decrease in AUC of the evaluation of venous invasion by HR-MRI. In addition, the evaluation criteria had a greater impact on CT than on MR. Further details are displayed in Table 4. The results of GDA evaluation are listed in Supplementary Table 2. The HR-MRI and non-HR-MRI cases are shown in Figs. 2 and 3.

**Table 4:** Predictive efficacy of each group under two assessment criteria

		A			V			A + V		
		Sen	Spe	AUC	Sen	Spe	AUC	Sen	Spe	AUC
C1	Group1	0.79 (11/14)	0.99 (298/302)	0.89	0.86 (12/14)	0.94 (61/65)	0.90	0.82 (23/28)	0.98 (359/367)	0.90
	Group2	0.67 (6/9)	0.98 (213/218)	0.82	0.46 (5/11)	0.98 (46/47)	0.72	0.55 (11/20)	0.98 (259/265)	0.76
	Group3	0.40 (4/10)	0.98 (293/298)	0.69	0.44 (8/18)	0.93 (55/59)	0.69	0.43 (12/28)	0.97 (348/357)	0.70
	Group4	0.50 (3/6)	0.98 (225/230)	0.74	0.64 (9/14)	0.96 (43/45)	0.80	0.60 (12/20)	0.97 (268/275)	0.79
C2	Group1	0.86 (12/14)	0.98 (296/302)	0.92	0.86 (12/14)	0.88 (57/65)	0.87	0.86 (24/28)	0.96 (353/367)	0.91
	Group2	0.78 (7/9)	0.97 (211/218)	0.87	0.73 (8/11)	0.96 (45/47)	0.84	0.75 (15/20)	0.97 (256/265)	0.86
	Group3	0.40 (4/10)	0.97 (289/298)	0.68	0.56 (10/18)	0.81 (48/59)	0.69	0.50 (14/28)	0.94 (337/357)	0.72
	Group4	0.50 (3/6)	0.97 (224/230)	0.74	0.79 (11/14)	0.93 (42/45)	0.86	0.70 (14/20)	0.97 (266/275)	0.83

C1, Criteria 1 (and); C2, Criteria 2 (or); A, artery; V, venous; Sen, sensitivity; Spe, specificity; AUC, area under the ROC curve; HR, high-resolution; ROC, Receiver operating characteristic; Group 1, HR-MRI group; Group 2, CT of HR-MRI group; Group 3, non-HR-MRI group; Group 4, CT of non-HR-MRI group

### Delong test between different groups and different criteria

The *P* values and *Q* values of the DeLong test for comparing the performance of different examination modalities or criteria are shown in Table 5. In the HR-MRI group, the C1 criteria were superior to C2 criteria in the assessment of venous invasion. HR-MRI was superior to CT in assessing venous and arteriovenous invasion according to the C1 criteria.

**Table 5:** P values and Q values of DeLong test between different groups

			A		V		A + V		
			P value	Q value	P value	Q value	P value	Q value	
Group1&2	Group1(C1vsC2)		0.3653	0.4384	0.0405	0.1215	0.5939	0.5939	
	Group2(C1vsC2)		0.3587	0.4384	0.0775	0.186	0.04003	0.1215	
	C1(Group1vs2)		0.1232	0.2464	0.007401	0.04441	0.001417	0.017	
	C2(Group1vs2)		0.2806	0.4209	0.4617	0.5037	0.1724	0.2955	
Group3&4	Group3(C1vsC2)		0.04441	0.1776	0.9312	0.9312	0.4203	0.5044	
	Group4(C1vsC2)		0.3173	0.4231	0.2257	0.4231	0.1791	0.231	
	C1(Group3vs4)		0.3173	0.4231	0.178	0.4231	0.2547	0.4231	
	C2(Group3vs4)		0.6553	0.7149	0.00535	0.0642	0.04223	0.1776	
MRI		C1(Group1vs3)	0.05131	0.05131	0.01023	0.02046	0.001102	0.004863	
		C2(Group1vs3)	0.01441	0.02162	0.0315	0.0378	0.001621	0.004863	
CT		C1(Group2vs4)	0.5525	0.8288	0.4321	0.7778	0.7712	0.8549	
		C2(Group2vs4)	0.31	0.7778	0.8549	0.8549	0.7343	0.8549	
Group2&4		C1vsC2	0.3686	0.7778	0.03188	0.1435	0.02921	0.1435	
Group2 C2vsGroup1 C1			0.2634	0.3161	0.3305	0.3305	0.1425	0.2685	
Group4 C2vsGroup3 C1			0.179	0.2685	0.006138	0.03683	0.01508	0.04524	

C1, Criteria 1 (and); C2, Criteria 2 (or); A, artery; V, venous; Sen, sensitivity; Spe, specificity; Group1, HR-MRI group; Group2, CT of HR-MRI group; Group3, non-HR-MRI group; Group4, CT of non-HR-MRI group

In the non-HR-MRI group, the C1 criterion was superior to the C2 criterion in assessing arterial invasion. CT was superior to non-HR-MRI in the assessment of venous and arteriovenous invasion according to C2 criteria, but not significant after FDR correction. HR-MRI was superior to non-HR-MRI for all vessels, regardless of the evaluation criteria.

The C1 and C2 criteria showed no difference on CT evaluation between the HR-MRI and non-HR-MRI groups. After combining the CT data from groups 2 and 4, the C2 criteria were found to be superior to the C1 criteria in the assessment of venous and arteriovenous invasion (0.85 vs. 0.79, *P* = 0.029). However, the differences were not statistically significant after FDR correction.

After applying different assessment criteria in different examination methods, CT assessment using the C2 criteria was superior to non-HR-MRI using the C1 criteria in the assessment of the venous and arteriovenous veins. The results of CT evaluation using the C2 criteria did not differ significantly from those of HR-MRI evaluation using the C1 criteria. Therefore, the C2 criteria play an important role in the diagnostic efficacy of CT evaluation.

### Combined HR-MRI and CT

To further evaluate the diagnostic efficacy of the combination of HR-MRI and CT, the presence of vascular invasion in the combined model was defined as a positive diagnosis using MR or CT. The C2 criteria were used for CT diagnosis, while both the C1 and C2 criteria were compared for MRI diagnosis. Patients without CT images were excluded when the two datasets were merged. Among the remaining 58 cases, the C1 and C2 criteria showed no difference in the arterial evaluation, whereas C1 showed better performance in the venous evaluation. This combination significantly improved the sensitivity of venous evaluation. A comparison of the combined and single-modality models revealed that although the combined model had higher overall effectiveness values, only the difference in the CT evaluation was significant. The detailed efficacy rates and comparisons are shown in Tables 6 and 7.

**Table 6:** Predictive efficacy of combined models

	A			V			A + V		
	Sen	Spe	AUC	Sen	Spe	AUC	Sen	Spe	AUC
HR-MRI C1 & CT C2	0.89	0.97	0.93	1.00	0.92	0.96	0.95	0.96	0.96
h-MRI C2 & CT C2	0.89	0.97	0.93	1.00	0.87	0.94	0.95	0.95	0.95

C1, Criteria 1 (and); C2, Criteria 2 (or); A, artery; V, venous; Sen, sensitivity; Spe, specificity

**Table 7:** Comparison of predictive efficacy of combined and single models

	versus	A	V	A + V
HR-MRI C1 & CT C2	HR-MRI C1	0.08188	0.6132	0.5344
	Group2 C2	0.3173	0.1098	0.03614
	h-MRI C2 & CT C2	1.00	0.1528	0.1565
h-MRI C2 & CT C2	HR-MRI C2	0.1564	0.6132	0.4863
	Group2 C2	0.3173	0.201	0.04427

C1, Criteria 1 (and); C2, Criteria 2 (or); A, artery; V, venous; Group2, CT of HR-MRI group

## Discussion

In recent years, HR-MRI has emerged to display the extent and characteristics of tumours, providing additional valuable information and assisting in treatment decisions [25–27]. Our study showed that gadobutrol-enhanced HR-MRI had higher sensitivity and specificity in detecting vascular invasion than conventional MRI and CT, and its performance was significantly better than that of non-HR-MRI. Better diagnostic results were obtained when HR-MRI and CT were combined to diagnose vascular invasion. Although the difference between the combination and HR-MRI findings was not significant, they showed markedly better results than CT. Therefore, preoperative HR-MRI combined with CT can be the preferred examination option for the diagnosis of vascular invasion.

In this study, the specificity of both CT and MRI evaluations was significantly better than their sensitivity, particularly for arterial evaluation. The possible reasons for this are as follows: First, the gold standard for determining vascular invasion is surgical exploration and pathology, with preoperative imaging determining resectable cases. In these cases, most of the evaluated vessels were spatially distant from the tumours and did not invade them. Second, the arterial diameter is smaller than that of the vein, with a more complex surrounding structure, affecting the observation of the vascular wall and the true positive rate. Third, the small sample size cannot be ignored, and a larger sample needs to be included for validation. Cases of arterial invasion are more difficult to treat because resection and reconstruction could be applied for venous invasion, whereas arterial reconstruction is extremely difficult [28, 29].

When comparing the imaging modalities for the evaluation of venous invasion, the specificity of CT was slightly higher than that of MR, whereas the sensitivity of HR-MRI was higher than that of the other methods. This might be because CT has better spatial resolution and insufficient soft tissue resolution, which is advantageous for observing the spatial tumour–vascular relationship. However, when an inflammatory exudate is present around the tumour, it is difficult to distinguish it from tumour infiltration using CT, resulting in an increased false-positive rate on CT evaluation [30–32]. HR-MRI accurately showed tumour margins and vessel wall structures with high soft tissue resolution, thus improving diagnostic accuracy.

When comparing the diagnostic criteria, the reason for the improved accuracy with C2 was mainly due to the improved sensitivity after broadening the diagnostic criteria for vascular invasion. However, the C2 criteria did not significantly improve the sensitivity of HR-MRI on venous evaluation but markedly reduced the specificity. This indicates that HR-MRI can accurately determine vascular integrity, but the use of tumour–vascular contact to determine vascular invasion can increase the false-positive rate. In particular, in non-HR-MRI, the C1 criterion showed better performance on arterial evaluation with high specificity. Although the C2 criterion slightly improved the AUC values for the overall vascular assessment in both MRI groups, the difference was not significant. In contrast, assessment of the arterial wall by MRI remains slightly limited owing to the slimmer diameters of the arteries than that of the veins. In the combined assessment of HR-MRI and CT, the C2 criteria did not have a significant impact on the overall MRI evaluation but rather reduced the specificity of the venous assessment. Therefore, the C1 criteria is more applicable to MR assessment than the C2 criteria.

Furthermore, the C2 criteria had a greater impact on the diagnostic efficacy of CT than that of MRI, especially for venous imaging. The C2 criterion significantly improved the sensitivity of the assessment but had little impact on specificity. This may be because CT has a better advantage in showing the vascular–tumour contact, but it does not directly and accurately show the vascular integrity. On expanding the invasion criteria to consider only tumour–vessel contact, the false-positive rate did not have a significant impact on the efficacy of CT. This indicates that the probability of vascular invasion is extremely high when the tumour encases the vessel by > 180 ° on CT. Therefore, C2 is more applicable than C1 for CT evaluation of vascular invasion.

Using CT alone is challenging for the assessment of vascular involvement, particularly after neoadjuvant therapy, owing to the confounding effects of therapy-related changes such as fibrosis and oedema [33, 34]. Despite many patients having suspicious imaging findings, the true need for vascular resection and venous invasion is relatively low, suggesting that vascular involvement is often overestimated using current imaging techniques. Therefore, it is necessary to analyse and evaluate the vascular invasion of tumours by combining various imaging methods such as CT and MRI.

Our study has several limitations. First, owing to the retrospective design of this study, it was difficult to draw broad conclusions regarding this patient population based on our results alone. Only patients with PDAC who were preoperatively judged operable were included in this study. We used surgical exploration and pathology as the gold standards for assessing vascular invasion. Second, the number of cases was limited, particularly because the number of patients with arterial invasion was small. Tumours defined as definitely unresectable on preoperative imaging, but not verified, were not included. Therefore, the relative percentage of patients with equivocal signs of vascular involvement may have increased, and the number of involved vessels may have decreased, lowering the sensitivity of CT and MR for detecting vascular involvement. Third, the combination of HR-MRI and gadobutrol showed better performance than conventional MRI with traditional gadolinium contrast agents. However, the weights of specific factors must be studied further. Finally, this study was conducted at a single academic institution using HR-MRI data obtained from a single MRI scanner. Data from more centres and scanners are required for further research and validation.

In conclusion, HR-MRI provides a more accurate assessment of PDAC vascular invasion than conventional MRI. CT and MRI may not share the same evaluation criteria because CT requires a loose standard to achieve accurate diagnosis. Preoperative HR-MRI combined with CT is recommended to obtain accurate vascular invasion assessments and assist in surgical treatment decision making.

### Electronic supplementary material

Below is the link to the electronic supplementary material.

Additional File 1: Non-HR-MRI scan sequence and parameters.

Additional File 2: Assessment of GDA invasion

## Data Availability

The datasets generated and analyzed during the current study are not publicly available due to participant privacy and ethical restrictions, but are available from the corresponding author on reasonable request.

## Abbreviations

PDAC: Pancreatic ductal adenocarcinoma
NCCN: National Comprehensive Cancer Network
CT: Computed tomography
MRI: Magnetic Resonance Imaging
HR-MRI: High-resolution MRI
CA: Celiac trunk artery
HA: Hepatic artery
CA: Celiac trunk artery
HA: Hepatic artery
SMA: Superior mesenteric artery
SpA: Splenic artery
PV-SMV: Portal vein-superior mesenteric vein
C1: Criteria 1
C2: Portal vein-superior mesenteric vein
ROC: Receiver operating characteristic
Sen: Sensitivity
Spe: Specificity
AUC: Area under the ROC curve
