# Supplementary material for: Preoperative assessment of peripheral vascular invasion of pancreatic ductal adenocarcinoma based on high-resolution MRI
Source: BMC Cancer. 2023 Nov 10;23:1092. doi: 10.1186/s12885-023-11451-8 (PMC10638695; doi:10.1186/s12885-023-11451-8)
Supplement: Supplementary file 1 — Additional File 1: Non-HR-MRI scan sequence and parameters. [file 12885_2023_11451_MOESM1_ESM.docx]

Supplementary Table 1. non-HR-MRI scan sequence and parameters

| Sequences | TR(ms) | TE(ms) | FOV(cm) | Scan matrix | Slice thickness (mm) | Flip angle (°) | Fat saturation |
| --- | --- | --- | --- | --- | --- | --- | --- |
| DIXON | 1.23 | 3.97 | 38 | 288×176 | 6 | 9 | No |
| T2WI | 2000 | 77 | 38 | 384×288 | 5 | 150 | No |
| T2WI-FS | 2000 | 77 | 38 | 384×288 | 5 | 103 | SPAIR |
| 3D_T1_VIBE | 2.75 | 1.05 | 38 | 320×192 | 2 | 12.5 | Q-fat |
| DWI* | 3500 | 47 | 38 | 134×100 | 6 | - | SPAIR |

TR, repetition time; TE, echo time; FOV, field of view; WI, weighted imaging; FS, fat-suppressed; VIBE, Volumetric Interpolated Body Examination; DWI, Diffusion-weighted imaging.

*b factors = 0, 50, 400 and 800 s/mm^2^.
