# Supplementary material for: Preoperative assessment of peripheral vascular invasion of pancreatic ductal adenocarcinoma based on high-resolution MRI
Source: BMC Cancer. 2023 Nov 10;23:1092. doi: 10.1186/s12885-023-11451-8 (PMC10638695; doi:10.1186/s12885-023-11451-8)
Supplement: Supplementary file 2 — Additional File 2: Assessment of GDA invasion [file 12885_2023_11451_MOESM2_ESM.docx]

Supplementary Table 2. Assessment of GDA invasion

| GDA | C1 | | | C2 | | |
| --- | --- | --- | --- | --- | --- | --- |
|  | All | Pancreas head/neck | Pancreas body/tail | All | Pancreas head/neck | Pancreas body/tail |
| Group1 | 27/79 | 19/50 | 8/29 | 34/79 | 23/50 | 11/29 |
| Group2 | 20/58 | 15/36 | 5/22 | 23/58 | 17/36 | 6/22 |
| Group3 | 19/77 | 19/53 | 0/23 | 24/77 | 24/53 | 0/23 |
| Group4 | 11/59 | 11/42 | 0/17 | 13/59 | 13/42 | 0/17 |

GDA, Gastroduodenal artery; Group 1, HR-MRI group; Group 2, CT of HR-MRI group; Group 3, non-HR-MRI group; Group 4, CT of non-HR-MRI group.
